# Neuroanatomical abnormalities in first-episode psychosis across independent samples: a multi-centre mega-analysis

**DOI:** 10.1017/S0033291719003568

**Published:** 2021-01

**Authors:** Sandra Vieira, Qiyong Gong, Cristina Scarpazza, Su Lui, Xiaoqi Huang, Benedicto Crespo-Facorro, Diana Tordesillas-Gutierrez, Víctor Ortiz-García de la Foz, Esther Setien-Suero, Floor Scheepers, Neeltje E.M. van Haren, René Kahn, Tiago Reis Marques, Simone Ciufolini, Marta Di Forti, Robin M Murray, Anthony David, Paola Dazzan, Philip McGuire, Andrea Mechelli

**Affiliations:** 1 Department of Psychosis Studies, Institute of Psychiatry, Psychology & Neuroscience, King's College London, London, UK; 2 Huaxi MR Research Center (HMRRC), Department of Radiology, West China Hospital of Sichuan University, Chengdu, China; 3 Psychoradiology Research Unit of Chinese Academy of Medical Sciences, West China Hospital of Sichuan University, Chengdu, Sichuan, China; 4 Department of Radiology, Shengjing Hospital of China Medical University, Shenyang, Liaoning, China; 5 Department of General Psychology, University of Padova, Padova, Italy; 6 CIBERSAM, Centro Investigación Biomédica en Red de Salud Mental, Madrid, Spain; 7 Department of Psychiatry, University Hospital Marqués de Valdecilla, School of Medicine, University of Cantabria-IDIVAL, Santander, Spain; 8 Neuroimaging Unit, Technological Facilities, Valdecilla Biomedical Research Institute IDIVAL, Santander, Cantabria, Spain; 9 Brain Centre Rudolf Magnus, University Medical Centre Utrecht, Utrecht, The Netherlands; 10 Social, Genetic & Developmental Psychiatry Centre, Institute of Psychiatry, Psychology & Neuroscience, King's College London, London, UK; 11 UCL Institute of Mental Health, University College London, UK

**Keywords:** First-episode psychosis, mega-analysis, multi-centre, neuroanatomy, voxel-based morphometry

## Abstract

**Background:**

Neuroanatomical abnormalities in first-episode psychosis (FEP) tend to be subtle and widespread. The vast majority of previous studies have used small samples, and therefore may have been underpowered. In addition, most studies have examined participants at a single research site, and therefore the results may be specific to the local sample investigated. Consequently, the findings reported in the existing literature are highly heterogeneous. This study aimed to overcome these issues by testing for neuroanatomical abnormalities in individuals with FEP that are expressed consistently across several independent samples.

**Methods:**

Structural Magnetic Resonance Imaging data were acquired from a total of 572 FEP and 502 age and gender comparable healthy controls at five sites. Voxel-based morphometry was used to investigate differences in grey matter volume (GMV) between the two groups. Statistical inferences were made at *p* < 0.05 after family-wise error correction for multiple comparisons.

**Results:**

FEP showed a widespread pattern of decreased GMV in fronto-temporal, insular and occipital regions bilaterally; these decreases were not dependent on anti-psychotic medication. The region with the most pronounced decrease – gyrus rectus – was negatively correlated with the severity of positive and negative symptoms.

**Conclusions:**

This study identified a consistent pattern of fronto-temporal, insular and occipital abnormalities in five independent FEP samples; furthermore, the extent of these alterations is dependent on the severity of symptoms and duration of illness. This provides evidence for reliable neuroanatomical alternations in FEP, expressed above and beyond site-related differences in anti-psychotic medication, scanning parameters and recruitment criteria.

## Introduction

Neuroanatomical abnormalities in schizophrenia have been well documented for the past four decades (Bora et al., 2011; Glahn et al., 2008). While the initial research was performed in patients with long-term schizophrenia (Ellison-Wright, Glahn, Laird, Thelen, & Bullmore, 2008), more recent studies have focussed on individuals in the early stages of the illness, when the effects of chronicity (Olabi et al., 2011; Vita, De Peri, Deste, & Sacchetti, 2012) and anti-psychotic medication (Radua et al., 2012; Shah et al., 2017; Vita, De Peri, Deste, Barlati, & Sacchetti, 2015) are minimal. The results of these studies, however, tend to be inconsistent from one investigation to another (Gao et al., 2018; Radua et al., 2012; Shah et al., 2017). For example, reports of insular abnormalities have been heterogeneous, with some studies reporting increased (Ren et al., 2013; Salgado-Pineda et al., 2003) and others decreased (Chua et al., 2007; Jayakumar, Venkatasubramanian, Gangadhar, Janakiramaiah, & Keshavan, 2005; Venkatasubramanian, 2010) grey matter volume (GMV) in this region. A possible explanation for these inconsistencies is that most studies have used small sample sizes and therefore may have been under-powered. For example, in the most recent meta-analyses (Gao et al., 2018; Radua et al., 2012; Shah et al., 2017), out of a total of 37 studies included (after accounting for overlapping studies across meta-analyses), 20 had a total sample size of 60 or less. Studies with small sample sizes are likely to result in overestimates of effect size and low reproducibility due to low statistical power (Button et al., 2013); which suggests that some of these small studies may have suffered from an increased risk of false positives. In addition to being under-powered, different studies have also varied significantly in terms of their methods such as recruitment criteria, imaging acquisition parameters, pre-processing and statistical analysis (Radua et al., 2012). Furthermore, the vast majority of studies have examined participants from a single research site, raising the possibility that the results might be specific to the characteristics of the local sample investigated.

To overcome some of these limitations, the ENIGMA consortium developed a standardized pipeline detailing data pre-processing and analysis procedures; once data are analysed, single-site results are pooled and summarized in a meta-analysis. This approach has led to unprecedented sample sizes in schizophrenia research, with two recent studies of cortical abnormalities in 4474 patients and 5098 controls (van Erp et al., 2018), and subcortical changes in a smaller, albeit still impressive, sample of 2028 patients and 2540 controls (van Erp et al., 2016). However, although this approach mitigates some of the main limitations of the traditional meta-analysis by reducing the heterogeneity of the pooled single studies, findings still rely on reported results from individual studies, which may result in limited accuracy (Shah et al., 2017). Multi-centre mega-analyses, involving the pre-processing and integration of data from independent studies in one single statistical analysis, provide an opportunity to overcome this limitation. Gupta et al. (2014) analysed neuroanatomical abnormalities in the first mega-analysis in schizophrenia in a sample comprised of 784 individuals with established schizophrenia and 936 healthy controls (HC) collected from 23 sites. More recently, Rozycki et al. (2018) analysed data from five sites totalling 448 HC and 387 patients with chronic schizophrenia. Similar mega-analytic efforts focussed on the initial stages of the illness, when the effects of confounders are minimal, are still non-existent and evidence is still reliant on small-to-modest sized studies (Gao et al., 2018; Shah et al., 2017).

In light of the limitations of the existing literature, the aim of this study was to use a multi-centre mega-analytic approach to test for neuroanatomical changes in first-episode psychosis (FEP) that are consistent across independent samples. Based on the findings of the recent meta-analyses (Gao et al., 2018; Radua et al., 2012; Shah et al., 2017), we hypothesize that (i) patients would show GMV decrease in a distributed bilateral network including fronto-temporal and insular areas, consistently across the five independent samples; (ii) given the previous reports of symptom-dependent neuroanatomical alterations in psychosis (Fusar-Poli, Radua, McGuire, & Borgwardt, 2012; Tang et al., 2012), these decreases would be more pronounced in patients with more severe symptoms; and (iii) consistent with the existing evidence of progressive neuroanatomical changes in psychosis (Olabi et al., 2011; Vita et al., 2012), these decreases would be more pronounced in patients with longer duration of illness.

## Methods

### Subjects

A total of 1074 participants were included in the analysis. The total sample comprised of data collected from FEP patients and HC recruited as part of five independent studies, from four sites, all of which were previously published: Chengdu (China) (Gong et al., 2015), London (England) (GAP study; Di Forti et al., 2009), Santander (Spain) (PAFIP study; Pelayo-Terán et al., 2008) and Utrecht (The Netherlands) (GROUP study; Korver, Quee, Boos, Simons, & de Haan, 2012). Below is a description of the recruitment criteria for each study. All patients were experiencing their first psychotic episode, defined as the first manifestation of psychotic symptoms meeting criteria for a psychotic disorder, as specified by the DSM-IV (APA, 2000) or ICD-10 (WHO, 2004). For each of the five sites, ethical approval was granted from the relevant Ethics Committees, and written informed consent was obtained from all participants. Demographic and clinical data for patients and HC within each site are summarized in Table 1.

**Table 1.** Demographic and clinical characteristics for FEP and HC for each site and total sample

		Chengdu, China (*N* = 240)		London, England (*N* = 168)		Santander A, Spain (*N* = 257)		Santander B, Spain (*N* = 223)		Utrecht, The Netherlands (*N* = 186)		Total (*N* = 1074)	
		HC	FEP	HC	FEP	HC	FEP	HC	FEP	HC	FEP	HC	FEP
*N*		118	122	92	76	113	144	78	145	101	85	502	572
Gender (%)	M	56 (48)	55 (45)	37 (40)	41(54)	70 (62)	81 (61)	48 (61)	89 (61)	69 (68)	68(80)	280 (56)	341 (60)
	F	62 (52)	67 (55)	55 (60)	35 (46)	43 (38)	56 (39)	30 (39)	56 (39)	32 (32)	17 (20)	222 (44)	231 (40)
		χ^2^ = 0.1, ns		χ^2^ = 3.2, ns		χ^2^ = 0.0, ns		χ^2^ = 0.0, ns		χ^2^ = 3.2, ns		χ^2^ = 1.7, ns	
Age M (s.d.)		25.8 (8.0)	27.0 (7.3)	26.5 (6.5)	27.0 (6.8)	29.7 (7.7)	29.3 (8.1)	28.0 (7.4)	29.5 (8.7)	26.8 (8.2)	25.4 (5.9)	27.8 (7.5)	27.7 (8.0)
		*t* = 1.8, ns		*t* = 0.4, ns		*t* = 0.5, ns		*t* = 1.4, ns		*t* = 1.3, ns		*t* = 0.1, ns	
TIV (L) M (s.d.)		1.5 (0.1)	1.5 (0.2)	1.5 (0.1)	1.5 (0.2)	1.5 (0.1)	1.4 (0.2)	1.5 (0.1)	1.5 (0.2)	1.5 (0.1)	1.5 (0.2)	1.5 (0.1)	1.5 (0.2)
		*t* *=* 0.9, ns		*t* = 0.4, ns		*t* = 0.7, ns		*t* = 0.2, ns		*t* = 0.4, ns		*t* = 0.8, ns	
Positive symptoms M (s.d.)		–	24.5 (6.9)^a^	–	13.7 (5.5)^a^	–	14.3 (4.4)^b^	–	13.5 (4.3)^b^	–	15.8 (6.3)^a^	–	–
Negative symptoms M (s.d.)		–	18.6 (8.6)^a^	–	15.7 (6.0)^a^	–	6.2 (5.0)^c^	–	6.2 (5.0)^c^	–	16.2 (6.9)^a^	–	–
Duration of illness (yrs) Med (IQR)		–	0.3 (0.9)	–	1.1 (1.3)	–	0.4 (0.7)	–	0.3 (1.0)	–	0.6 (1.4)	–	0.4 (0.9)
Antipsychotic medication (N) (naïve/typical/atypical/NA)		–	122/0/0/0	–	7/2/56/11	–	2/0/142/0	–	2/20/116/7	–	0/3/48/34	–	133/25/362/52

TIV, total intra-cranial volume; L, litres; M, male; F, female; FEP, first-episode psychosis; HC, healthy controls; Med, median; M, mean; s.d., standard deviation; NA, not available.

a PANSS: Positive and Negative Symptoms Scale.

b SAPS: Scale for the Assessment of Negative Symptoms.

c SANS: Scale for the Assessment of Negative Symptoms.

ns: *p* > 0.05.

#### Site1: Chengdu, China

First-episode patients were recruited from the West China Hospital of Sichuan University, Chengdu, China as part of a wider study of psychiatric disorder diagnosis. Diagnosis of first episode of schizophrenia was determined by the consensus of two clinical psychiatrists using the Structured Interview for the DSM-IV Axis I Disorder (SCID) (First, Gibbon, Spitzer, & Williams, 1997). At the time of scanning, all patients were medication-naïve. HC were recruited by poster advertisement and screened using the SCID-I to confirm the lifetime absence of psychiatric disorders, as well as interviewed and subsequently excluded if they had any known history of psychiatric illness in first-degree relatives. Participants were excluded if they met any of the following criteria: (i) history of drug or alcohol abuse, (ii) pregnancy, and (iii) any physical illness such as hepatitis, cardiovascular disease or neurological disorder, as assessed by interview and review of medical records.

#### Site 2: London, England

Participants were recruited from the South London and Maudsley Foundation Trust and scanned at the Institute of Psychiatry, Psychology and Neuroscience. All patients meeting ICD-10 criteria for a diagnosis of psychosis (codes F20–F29 and F30–F33) (World Health Organization, 2004) were invited to participate in the study; patients with a diagnosis of organic psychosis were later excluded. HC were recruited through local advertisement from the same geographical areas as patients. A screening tool (Psychosis Screening Questionnaire; Bebbington & Nayani, 1995) was used to exclude the presence of psychotic symptomatology or a history of psychotic illness in controls. Additional exclusion criteria for all participants included learning disabilities (based as an IQ < 70), current or past neurological illness, brain injury with the loss of consciousness for more than 1 h and suspected or confirmed pregnancy.

#### Sites 3 and 4: Santander A and B, Spain

Data from Santander A and B were acquired as part of the same large prospective longitudinal study on the first non-affective episode psychosis in Cantabria, although with two different scanners. Individuals with FEP were recruited from both inpatient units and community mental health care centres. Patients were included if they met the following criteria: (1) age 15–60 years; (2) DSM-IV criteria for a principal diagnosis of schizophrenia, schizophreniform disorder, schizoaffective disorder, brief reactive psychosis or not otherwise specified psychosis; and (3) no prior treatment with anti-psychotic medication or, if previously treated, a total life time of adequate anti-psychotic treatment of <6 weeks. Patients with DSM-IV diagnoses of mental retardation or substance dependence (except nicotine dependence) were excluded. Age- and gender-matched HC were recruited from the community through advertisements and were screened for current or past history of psychiatric, mental retardation, neurological or general medical illnesses, including substance dependence and significant loss of consciousness, as determined by using an abbreviated version of the Comprehensive Assessment of Symptoms and History (CASH) (Andreasen, Flaum, & Arndt, 1992). The absence of psychosis in first-degree relatives was confirmed by clinical records and family interview.

#### Site 5: Utrecht, The Netherlands

Patients were identified through clinicians working in regional psychosis departments or academic centres and were included if they met the following criteria: (1) age range of 16–50 years; (2) a diagnosis of non-affective psychotic disorder according to the DSM-IV; (3) good command of the Dutch language; and (4) able and willing to give written informed consent. Controls were selected through a system of random mailings to addresses in the catchment areas of the cases and were included if the following criteria were met: (1) age range of 16 and 50 years, (2) no lifetime psychotic disorder, (3) no first-degree family member with a lifetime psychotic disorder, (4) good command of the Dutch language, and (5) able and willing to give written informed consent.

### Magnetic resonance imaging (MRI) data acquisition

At all five sites, volumetric MRIs were acquired using a T1-weighted protocol. At three sites, the scanner field strength was 3T, and at two sites, it was 1.5T. The details of the MRI acquisition sequence for each site can be found in the online Supplementary material sTable 1.

### Data analysis

#### Socio-demographic and clinical parameters

Differences between FEP and HC in gender, age and total intra-cranial volume (TIV) were assessed with a χ^2^ and independent-sample *t* test for categorical and continuous data, respectively, using SPSS v24.

#### Pre-processing

From the initial pool of 1249 images made available, 21 were excluded due to scanner artefacts and gross anatomical abnormalities, 71 due to excessive noise and a further 83 were excluded to keep the maximum and minimum age (18–55) the same across all sites. Differences in GMV between HC and FEP were examined using voxel-based morphometry (VBM), as implemented in SPM12 software (http://www.fil.ion.ucl.ac.uk/spm/software/spm12/) running under MATLAB 9 (The MathWorks, Inc, Natick, Massachusetts, USA) (Ashburner & Friston, 2005). The following steps were followed for the pre-processing of each site: (1) checking for scanner artefacts and gross anatomical abnormalities for each subject; (2) setting the anterior commissure as the origin of the stereotaxic space and reorienting the image along the anterior commissure–posterior commissure (AC–PC) line; and (3) segmenting the image into grey matter, white matter and CSF maps. Next, all available images were used to create a study-specific template as implemented by the DARTEL (diffeomorphic anatomical registration using exponentiated lie algebra) toolbox (Ashburner, 2007). This procedure warps the grey matter and white matter partitions into a new study-specific reference space representing an average of all the subjects included in the analysis, thus maximizing accuracy and sensitivity (Yassa & Stark, 2009). Finally, GMV maps were normalized to the Montreal Neurological Institute (MNI) template and subsequently smoothed with an 8 mm Gaussian filter. A ‘modulation step’ was also included in the normalization step to preserve the information about the absolute grey matter values (Mechelli, Price, Friston, & Ashburner, 2005). The final smoothed, modulated, normalized data were used for the statistical analysis.

To assess the reliability of our findings, the analysis pipeline described above was replicated using: (1) CAT12 toolbox (http://dbm.neuro.uni-jena.de/cat/), (2) a template built with a homogeneous (equal number of patients and controls across sites) sub-sample and (3) different size kernels for smoothing. Results are shown in the online Supplementary materials.

#### Statistical analysis

**Fig. 1.**
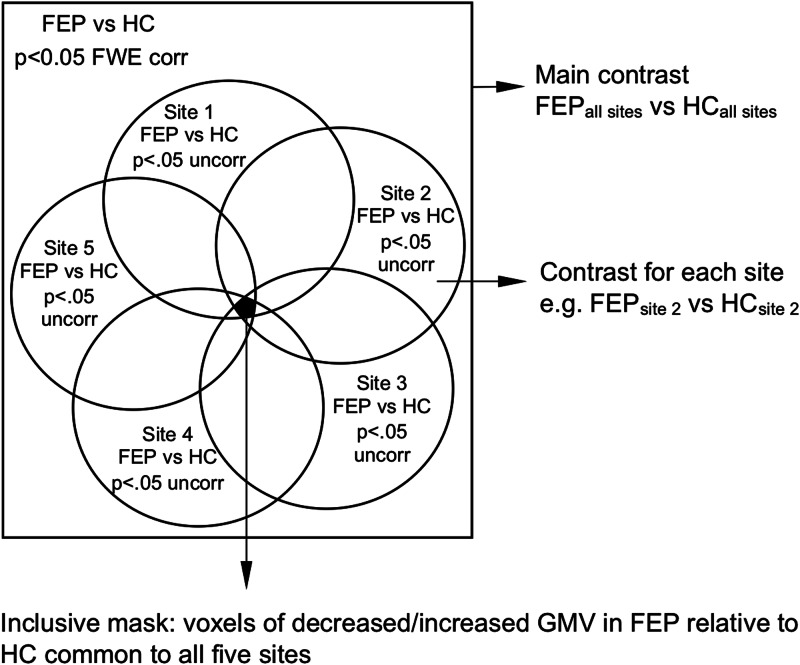
Inclusive masking procedure used to identify neuroanatomical abnormalities in FEP relative to HC consistent across all five sites. Left: an overall contrast with all FEP against all HC (*p* < 0.05 FWE corrected) was combined with five site-level contrasts (*p* < 0.05 uncorrected); this allowed us to identify only the voxels that survived both types of contrasts (intersection of all contrasts in black).

Statistical analysis was carried out using an analysis of variance, with diagnostic group and scanning site as factors, resulting in 10 experimental groups. Age and gender were included as covariates of no interest. The option of proportional scaling was selected to remove confounding driven by global differences. Neuroanatomical alterations in patients with FEP relative to HC consistent across the five datasets were identified using the ‘inclusive masking’ option as implemented in SPM software. This option allowed us to test for voxels which showed (i) an overall statistically significant difference between patients and HC across all sites (*p* < 0.05 FWE corrected) and (ii) at least a strong trend at each site (*p* < 0.05 uncorrected). Specifically, this consisted of the following steps in SPM: (i) comparing all FEP against all HC at *p* < 0.05 FWE corrected using an overall main contrast – FEP_all sites_ *v.* HC_all sites_ (e.g. FEP_all sites_ < HC_all sites_), (ii) overlaying this contrast with a second set of five FEP *v.* HC contrasts, one for each site (e.g. FEP_site 1_ < HC_site 1_) at *p* < 0.05 uncorrected each, and finally (iii) identifying voxels of increased/decreased GMV in FEP relative to HC that survived both the overall and the site-level contrasts (Fig. 1). This procedure ensured that any overall statistically significant difference across the five sites would also be present at each site, at least at trend level. Statistical inferences were made using a minimum extent threshold of 50 voxels.

The TIV for each image was estimated by first calculating the volume of grey matter, white matter and CSF separately at each voxel from the segmented images; the total volume for each type of tissue was then calculated by summing the respective voxel-level volumes; finally, TIV was obtained by adding the volume of all three tissue types. The effects of symptom severity, illness duration and anti-psychotic medication on the identified clusters were estimated using Pearson's correlation between the values of GMV for the peak coordinate of each statistically significant cluster and each one of the clinical variables of interest. The raw psychotic symptom severity scores (acquired with either PANSS or SANS/SAPS) were first normalized to ensure comparability across sites. This normalization was achieved using the following formula:
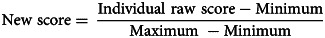
where Minimum and Maximum refer to the lowest and highest score allowed for either PANSS or SAPS/SANS. The resulting disease severity scores were scaled between 0 and 1. Across all sites (except site 1, where all patients were AP-naïve), AP medication dose was estimated by calculating the chlorpromazine equivalent (mg/day) for each individual according to Gardner, Murphy, O'Donnell, Centorrino, and Baldessarini (2010). Both chlorpromazine equivalent and duration of illness were log transformed. The statistical significance of Pearson's correlation was assessed using a *p* value<0.05 with Bonferroni correction for multiple comparisons.

## Results

### Socio-demographic and clinical parameters

There were no significant differences between FEP and HC in gender, age and TIV, both when considering all sites together and within each single site. Patients reported a comparable median duration of illness across sites (Table 1).

### Decreased GMV in FEP compared to HC

**Fig. 2.**
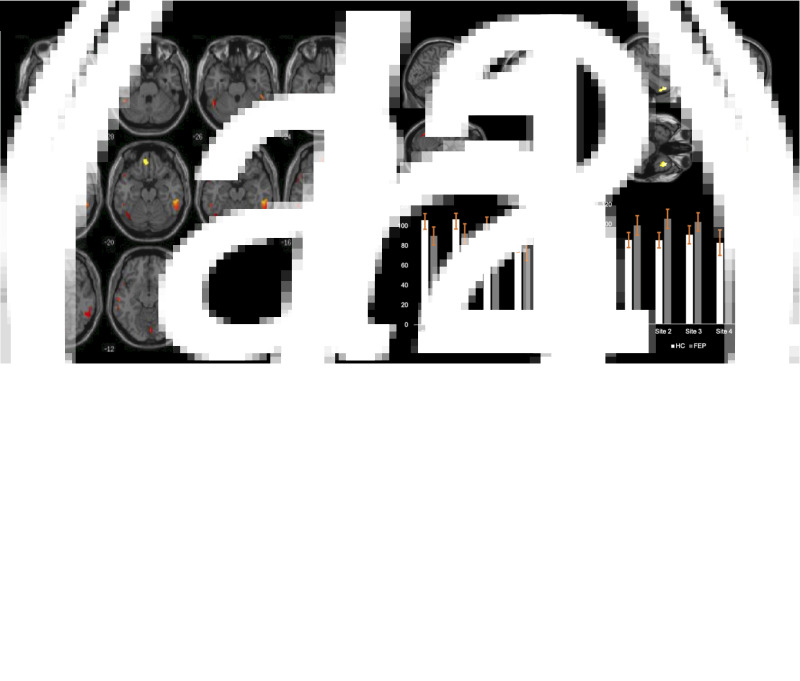
(*a*1) Regions showing statistically significant decreases in FEP relative to HC across the whole brain. (*a*2) Top Location of the gyrus rectus (straight gyrus) where the most pronounced GMV decrease was found; bottom: mean and standard deviation of the GMV in this region for each site. (*b*) Top Location of the right superior temporal gyrus (the only region showing statistically significant GMV increase in FEP relative to HC); bottom: mean and standard deviation of the GMV in this region for each site.

Relative to HC, FEP showed a widespread pattern of decreased GMV in fronto-temporal, insular and occipital regions bilaterally (see Table 2 and Fig. 2*a*1). The most pronounced GMV decrease was found in the left gyrus rectus, located in the inferior frontal lobe (Fig. 2*a*2; the mean-plots for the remaining significant clusters are shown in the online Supplementary material sFig. 1); negative correlations were found between GVM in this region and severity of both positive and negative symptoms. The right lingual gyrus also showed negative correlations with both positive and negative symptoms as well as with the duration of illness. In addition, negative correlations were found between both the left inferior temporal gyrus and the left fusiform gyrus and positive symptoms (Table 3). No significant associations were detected between any brain region and anti-psychotic medication (Table 3). Scatter plots for the significant correlations are reported in the online Supplementary material sFig. 2.

**Table 2.** MNI coordinates and *z* scores for regions showing GMV changes in FEP relative to the HC

Region	Peak MNI coordinates (*x*,*y*,*z*)	Cluster size (No of voxels)	*z*	*p*
Decreased GVM in FEP relative to HC				
L gyrus rectus	−6,34,−21	159	9.6	0.002
L med. orbital gyrus	−9,54,−15		8.4	
L sup. temporal pole	−21,8,−32	119	9.3	0.004
L fusiform gyrus	−20,2,−42		8.8	
R inf. temporal gyrus	56,−36,−20	607	9.1	<0.001
R mid. temporal gyrus	62,−32,−12		8.7	
L inf. temporal gyrus	−51,−52,−27	239	8.9	0.001
L fusiform gyrus	−46,−58, −22		8.6	
L mid. temporal gyrus	−58,−21,−14	161	8.8	0.002
R lingual gyrus	2,−80,−10	106	8.7	0.004
L mid. temporal gyrus	−52,−42,−18	63	8.5	0.009
L sup. temporal gyrus	−48, 18,−16	86	8.4	0.006
R insula	45,18,−8	88	8.3	0.006
Increased GVM in FEP relative to HC				
R sup. temporal gyrus	38,16,−38	338	8.1	<0.001

GMV, grey matter volume; FEP, first-episode psychosis; HC, healthy controls; L, left; R, right; med., medial; mid., middle; inf., inferior; sup., superior.

**Table 3.** Pearson's correlations between regions showing GMV changes in FEP relative to the HC and symptom severity, illness duration and anti-psychotic medication

	Positive symptoms	Negative symptoms	Duration of illness	Anti-psychotic medication
Decreased GVM in FEP relative to HC				
L gyrus rectus	**−0**.**31**	**−0**.**20**	−0.10	−0.08
L med. orbital gyrus	**−0.17**	**−0.17**	−0.03	−0.06
L sup. temporal pole	−0.06	−0.07	−0.09	−0.04
L fusiform gyrus	−0.05	−0.05	−0.11	0.02
R inf. temporal gyrus	−0.11	−0.04	−0.08	−0.04
R mid. temporal gyrus	0.04	0.07	−0.01	−0.02
L inf. temporal gyrus	**−0.17**	−0.13	−0.10	−0.11
L fusiform gyrus	**−0.15**	−0.12	−0.08	−0.09
L mid. temporal gyrus	0.09	0.08	0.06	0.02
R lingual gyrus	**−0.20**	**−0.16**	**−0.18**	−0.13
L mid. temporal gyrus	−0.07	−0.06	−0.11	−0.08
L sup. temporal gyrus	0.02	−0.04	−0.06	−0.03
R insula	0.03	−0.02	**−0.15**	−0.08
Increased GVM in FEP relative to HC				
R sup. temporal gyrus	0.05	0.07	0.02	−0.08

GMV, grey matter volume; FEP, first-episode psychosis; HC, healthy controls; L, left; R, right; med., medial; mid., middle; inf., inferior; sup., superior.

Statistical inferences were made at *p* < 0.05 after Bonferroni correction for multiple comparisons based on the number of regions; this resulted in a *p* value of 0.05/14 = 0.0035. Statistically significant correlations are shown in bold.

### Increased GMV in FEP compared to HC

A significant increase in GMV in FEP relative to HC was found in the right superior temporal gyrus (Table 2 and Fig. 2*b*). The volume of this region was not significantly associated with the severity of positive or negative symptoms, duration of illness or anti-psychotic medication (Table 3).

## Discussion

Most previous studies on the neuroanatomical basis of FEP have used small samples recruited within a single site, and have yielded heterogeneous findings (Gao et al., 2018; Radua et al., 2012; Shah et al., 2017). The aim of this study was to use a multi-centre mega-analytic approach to identify neuroanatomical changes in FEP that are expressed consistently across several independent studies. As hypothesized, we found a widespread bilateral pattern of GMV decrease in fronto-temporal, insular and occipital regions. Some of these effects, particularly in the orbitofrontal and lingual gyri, were correlated with symptom severity and duration of illness. In addition, an increase in GMV was found in the right superior temporal lobe. Critically, all patients were experiencing their first episode of psychosis and one of the five samples was medication-naïve. In what follows, we discuss the brain structures that emerged from this study as well as their main role in the psychopathology of the early stages of psychosis.

### Orbitofrontal cortex

A significant decrease in GMV was found in the two sub-regions of the orbitofrontal cortex (OFC), namely the gyrus rectus (straight gyrus) and the orbital gyrus (Buchanan et al., 2004; Nakamura et al., 2007). Grey matter deficits in the OFC have been reported in established psychosis (e.g. Kim, Kim, & Jeong, 2017; Kong et al., 2015; Rimol et al., 2012; Xu et al., 2017) and, to a lesser extent, in FEP (e.g. Crespo-Facorro, Kim, Andreasen, O'Leary, & Magnotta, 2000; Huang et al., 2015; Keymer-Gausset et al., 2018; Liao et al., 2015; Tordesillas-Gutierrez et al., 2015), consistent with the so-called ‘hypo-frontality’ hypothesis of psychosis; although increases in this region have also been observed (Gao et al., 2018). The OFC has been implicated in multiple functions, including cognitive flexibility, reward learning and decision making (see Kringelbach, 2005; Schoenbaum, Roesch, Stalnaker, & Takahashi, 2009 for a review), most of which are impaired in people with psychosis (Aas et al., 2014; Murray et al., 2008; Premkumar et al., 2015; Strauss, Waltz, & Gold, 2014). The gyrus rectus (straight gyrus) was the region with the most pronounced decrease in GMV within the OFC and the whole brain. Consistent with our finding, this region has been reported to be decreased in FEP regardless of anti-psychotic medication status in a recent meta-analysis (Shah et al., 2017). This is also consistent with the lack of a statistically significant association between this region and anti-psychotic medication found in the present study. A decrease in GMV in the gyrus rectus was also found in the largest single-site VBM study of first-episode patients to date which included 93 FEP participants and 175 controls (Meisenzahl et al., 2008); although evidence for normal volume has also been reported (Roiz-Santiáñez et al., 2011; Takayanagi et al., 2011). As hypothesized, GMV in the gyrus rectus was inversely related to positive and negative symptoms – consistent with previous studies (Kim et al., 2017; Sans-Sansa et al., 2013; Szendi et al., 2006).

### Insula

Despite inconsistences across individual studies, most of the existing literature indicates deficits in the insular cortex of people with FEP, albeit with some inconsistencies in the exact location of the effect (Crespo-Facorro, Kim, Andreasen, O'Leary, Bockholt, et al., 2000; Gao et al., 2018; Shah et al., 2017). In the present investigation, it was the anterior part of the insula that showed reduced GMV. This region plays an important role in salience processing (Menon & Uddin, 2010), emotional appraisal and social cognition (Eckert et al., 2009), all of which are affected in psychosis (Wylie & Tregellas, 2010). Notably, grey matter deficits in the insula, as well as in the gyrus rectus and superior temporal gyrus, have also been found in individuals at ultra-high risk for psychosis who later transitioned to psychosis (Smieskova et al., 2010); this suggests reduced GMV in this region may represent a neuroanatomical signature of vulnerability to psychosis rather than a marker of the actual illness. Furthermore, a GMV decrease in this region has been found to be expressed above and beyond ethnic variations in incidence and clinical expression (Gong et al., 2015).

### Temporal cortex

Decreased GMV in temporal regions are amongst the most replicated findings in psychosis, including in FEP (Chan, Di, McAlonan, & Gong, 2011; Radua et al., 2012; Shah et al., 2017). In this study, several temporal regions showed GMV deficits, namely the superior, middle and inferior gyri as well as the temporal portion of the fusiform gyrus bilaterally. GMV deficits in the left superior temporal gyrus are thought to play a central role in auditory verbal hallucinations in FEP patients (Benetti et al., 2015; Modinos et al., 2013), possibly due to the role of this region in language perception and processing; it has been suggested that impairment to this region may lead to a misattribution of internal speech (Frith & Done 1988; Mechelli et al., 2007). The fusiform gyrus is also thought to play an important role in the psychopathology of psychosis, mainly due to its contribution to facial recognition (Haxby, Hoffman, & Gobbini, 2000, 2002), which is impaired in psychosis (see Green, Horan, & Lee, 2015; Barkl, Lah, Harris, & Williams, 2014 for a review) and is often seen as a proxy for the social cognition deficits characteristic of the illness (Green et al., 2015). Perhaps more challenging to interpret is the significant increase in GMV in the right superior temporal gyrus. Nevertheless, increases in patients relative to controls across the brain, including the temporal cortex, have been reported before (Kim et al., 2003; Lee et al., 2011; Radewicz, Garey, Gentleman, & Reynolds, 2000; Taylor et al., 2005), and are typically interpreted in terms of a ‘compensatory mechanism’ (Guo, Palaniyappan, Liddle, & Feng, 2016) or a transient inflammation resulting from increased apoptotic activity during which apoptotic cells are removed (Adler, Levine, DelBello, & Strakowski, 2005; Berger, Wood, & McGorry, 2003).

### Lingual gyrus

Evidence supporting structural abnormalities in the lingual gyrus in FEP has not been as consistent, with some studies reporting decreased (Ellison-Wright et al., 2008) and others increased (Gao et al., 2018) GMV. Such inconsistency may be explained by medication status, as shown by Shah et al. (2017), where GMV of the lingual gyrus was decreased in anti-psychotic naive FEP patients but increased in FEP patients undergoing anti-psychotic treatment. However, in our study, which included both samples with and without exposure to anti-psychotics, there was a consistent GMV decrease in the lingual gyrus in each of the five sites, suggesting that a GMV decrease in this region may be present above and beyond medication status. Nevertheless, the lingual gyrus was significantly associated with anti-psychotic medication, therefore indicating that this region may be particularly prone to alterations when exposed to medication. The lingual gyrus is involved mainly in visual processing (Hahn, Ross, & Stein, 2006; Lee, Hong, Seo, Tae, & Hong, 2000) which has been shown to be impaired in psychosis (see Butler, Silverstein, & Dakin, 2008; Silverstein & Keane, 2011 for a review) and are also thought to underlie some of the cognitive impairments characteristic of the illness (Contreras, Tan, Lee, Castle, & Rossell, 2018; Surti & Wexler, 2012; Surti, Corbera, Bell, & Wexler, 2011). The lingual gyrus also contributes to the evaluation of emotional faces (Fusar-Poli et al., 2009) which, together with the deficits found in the fusiform gyrus, may explain social cognition impairments in psychosis (Green et al., 2015).

### Limitations

A first limitation of this study was that clinical data were acquired using different instruments (positive symptoms were assessed with either the PANSS or SAPS and negative symptoms with the PANSS or SANS). We overcame this limitation by normalizing individual scores within each scale as in the previous studies (Gong et al., 2019). The resulting scores were highly correlated (*r* = 0.87) with automated methods to convert scores between these two widely used scales (van Erp et al., 2014). A further limitation is that there were differences in age, gender and clinical presentation across the five samples. However, we do not think this undermines the reliability of our findings, since our statistical analysis tested for common neuroanatomical abnormalities across the five sites rather than site-specific effects. Additionally, the MRI data were not harmonized across sites using dedicated approaches such as ComBat (Johnson, Li, & Rabinovic, 2006), which could have improved the reliability of the results. The majority of VBM studies so far, including the present study, have used the DARTEL approach in-built in SPM to create study-specific templates. Although this is a well-established method, future studies could benefit from the use of recent alternative approaches, such as ANTs (http://stnava.github.io/ANTs/). A final limitation is that the five datasets differed with respect to anti-psychotic medication, with one sample being medication-naïve and the remaining four samples receiving various degrees of medication (Table 1). Critically, when we examined the impact of anti-psychotic medication on the findings, we found little evidence of statistically significant effects. This can be explained by the fact that our findings were based on the consistent neuroanatomical abnormalities across the five datasets, which included both medicated and non-medicated samples.

## Conclusion

This study aimed to overcome the limitations of small and single-site studies by conducting a multi-centre mega-analysis of neuroanatomical abnormalities in FEP. To the best of our knowledge, this is the largest VBM study in FEP to date. We found that a widespread pattern of fronto-temporal, insular and occipital decreased GMV in FEP that were expressed consistently across five independent studies; overall, these decreases were not affected by anti-psychotic medication. This provides evidence for reliable neuroanatomical alternations in FEP, expressed above and beyond site-related differences in anti-psychotic medication, scanning parameters and recruitment criteria. With the increasing availability of larger datasets, future multi-centre mega-analyses could investigate the diagnostic specificity of these findings by integrating the data collected from people with different psychiatric diagnoses (Ellison-Wright & Bullmore, 2010; Gong et al., 2019).

## Supplementary material

For supplementary material accompanying this paper visit https://doi.org/10.1017/S0033291719003568. click here to view supplementary material
