# Supplementary material for: Neuroanatomical abnormalities in first-episode psychosis across independent samples: a multi-centre mega-analysis
Source: Psychol Med. 2019 Dec 20;51(2):340–50. doi: 10.1017/S0033291719003568 (PMC7893510; doi:10.1017/S0033291719003568)
Supplement: Supplementary file 1 [file S0033291719003568sup001.docx]

**Neuroanatomical abnormalities in first episode psychosis across independent samples: a multi-centre mega-analysis**

**Supplementary materials**

Table of Contents

**sTable 1. Image acquisition parameters for each site…………………………………………………..2**

[1. Replication of the VBM analysis using CAT12 3](#_Toc16414995)

[sTable 2. MNI coordinates and z scores for regions showing GMV changes in FEP relative to the HC using CAT12. 3](#_Toc16414996)

[2. Replication of the VBM analysis using a template built with a homogeneous sample 4](#_Toc16414997)

[sTable 3. Demographic and clinical characteristics for the homogenous sample used to build the template. 4](#_Toc16414998)

[sTable 4. MNI coordinates and z scores for regions showing GMV changes in FEP relative to the HC using a template generated with a homogeneous sample. 5](#_Toc16414999)

[3. Replication of the VBM analysis using a different size kernel 6](#_Toc16415000)

s[Table 5. MNI coordinates and z scores for regions showing GMV changes in FEP relative to the HC using a 6mm kernel. 6](#_Toc16415001)

s[Table 6. MNI coordinates and z scores for regions showing GMV changes in FEP relative to the HC using a 10mm kernel. 7](#_Toc16415002)

[sFigure 1. Mean-plots for the GMV of the peak coordinates of each cluster (error bars represent standard deviation). 8](#_Toc16415003)

[sFigure 2. Scatter plots for the significant correlations in Table 4. 9](#_Toc16415004)

# sTable 1. Image acquisition parameters for each site.

|  | Site 1 | Site 2 | Site 3 | Site 4 | Site 5 |
| --- | --- | --- | --- | --- | --- |
| Field strength (T) | 3 | 3 | 3 | 1.5 | 1.5 |
| TR/TE (ms) | 6.9/ 2.8 | 8.5/3.4 | 8.2/3.7 | 24/5 | 30/4.6 |
| Slice thickness (mm) | 1.2 | 1 | 1 | 1.5 | 1.2 |
| Data matrix | 512x512x156 | 256x256x166 | 256x256x160 | 256x256x124 | 256x256x170 |
| Voxel size (mm) | 0.47x0.47x1 | 1.02x1.02x1.2 | 0.94x0.94x1 | 1.02x1.02x1.5 | 1x1x1.2 |

# 1. Replication of the VBM analysis using CAT12

***1.1. Method***

In order to assess the robustness of the findings, the same 1074 T1-weighted images were segmented using CAT12 toolbox (http://www.neuro.uni-jena.de/cat/), running on SPM 12 (http://www.fil.ion.ucl.ac.uk/spm/software/spm12/) running under MATLAB 9 (The MathWorks, Inc, Natick, Massachusetts). The resulting segmented grey and white matter images were used to build a study-specific template, and the warped images normalized to MNI space, using the same approach as described in the Methods section.

***1.2. Results***

## **sTable 2.** MNI coordinates and z scores for regions showing GMV changes in FEP relative to the HC using CAT12.

| Region | | Peak MNI Coordinates (x,y,z) | Cluster Size  (No of voxels) | z | *p* |
| --- | --- | --- | --- | --- | --- |
| Decreased GVM in FEP relative to HC | | |  |  |  |
| R thalamus | | 6,-20,3 | 558 | 9.4 | <.001 |
| L thalamus | | -3,-20,3 |  | 9.3 |  |
| L gyrus rectus | | -6,48,-22 | 110 | 9.3 | .003 |
| R insula | | 37,-9,0 | 88 | 9.2 | <.001 |
| L insula | | -38,-10,-3 | 103 | 9.1 | .002 |
| R inf. temporal gyrus | | 60,-34,-21 | 399 | 8.9 | <.001 |
| R fusiform gyrus | | 48,-56,-18 |  | 8.5 |  |
| R mid. temporal gyrus | | 62,-45,-18 |  | 8.4 |  |
| R frontal sup. orbital gyrus | | 15,30,-21 | 94 | 8.9 | .004 |
| R frontal mid. orbital gyrus | | 28,40,-16 |  | 7.9 |  |
| Increased GVM in FEP relative to HC | | |  |  |  |
| L cingulate gyrus | -6,-12,38 | | 89 | 8.3 | .005 |
| L cingulate gyrus | -9,-33,38 | | 443 | 8.3 | <.001 |
| L precuneus | -8,-57,24 | |  | 7.9 |  |
| R cingulate gyrus | 12,-34,39 | | 218 | 8.2 | .001 |
| R precuneus | 10,-46,51 | |  | 7.5 |  |
| L cerebellum | -30,-40,-34 | | 54 | 8.1 | .009 |
| R caudate | 38,-14,20 | | 58 | 8.1 | .008 |

GMV: grey matter volume; FEP: first episode psychosis; HC: healthy controls; L: left; R: right; med.: medial; mid.: middle; inf.: inferior; sup.: superior.

Lowering the extended threshold to 25 consecutive voxels added two more clusters of reduced GMV in FEP consistent with the original analysis: right gyrus rectus (MNI coordinates: 8,27,-21, cluster size: 29, z=9.3 , *p*=0.016) and right lingual gyrus (MNI coordinates: -6,80,6, cluster size: 29, z=8.0 , *p*=0.016). Overall, the same pattern of frontal-temporal-insular reductions was also detected using this approach. One additional cluster containing the right and left thalamus – was further identified; although not present in the original analysis, GMV decreases in this area have been well documented (van Erp *et al.*, 2016). The pattern of increased GMV, on the other hand, was not as consistent with the results of the main analysis; however, such increased has been found in previous large-scale studies and meta-analysis and may therefore represent a true difference (van Erp *et al.*, 2016, 2018; Gao *et al.*, 2018).

# 2. Replication of the VBM analysis using a template built with a homogeneous sample

***2.1. Method***

To ensure the results obtained were not affected by the fact that the study-specific template was built with a different number of participants at each site, the same VBM analysis was repeated after generating a template from a homogeneous sub-sample. The smallest group across sites was site 2 with 76 FEP participants. Therefore, the remaining FEP and HC groups for each site were randomly sampled for 76 participants, such that there were no significant differences in gender or age distribution between FEP and HC both at each site and in the total sample. The demographics of the final homogeneous sub-sample are shown in Table s2. Once generated, the template was used to normalize all 1074 grey matter segmented images, following the standard procedure described in section 2.3.2. Pre-processing in the main manuscript.

## **sTable 3.** Demographic and clinical characteristics for the homogenous sample used to build the template.

|  | | | Chengdu, China  (N=152) | | London, England  (N=152) | | Santander A,  Spain (N=152) | | Santander B,  Spain (N=152) | | Utrecht,  The Netherlands  (N=152) | | | Total  (N=760) | | |
| --- | --- | --- | --- | --- | --- | --- | --- | --- | --- | --- | --- | --- | --- | --- | --- | --- |
|  |  | | HC | FEP | HC | FEP | HC | FEP | HC | FEP | HC | FEP | HC | | FEP |  |
| N | | | 76 | 76 | 76 | 76 | 76 | 76 | 76 | 76 | 76 | 76 | 380 | | 380 |  |
| Gender (%) | | M | 36 (47) | 30 (39) | 31 (41) | 41  (54) | 46 (61) | 48 (63) | 46 (61) | 48 (63) | 53 (70) | 60(79) | 212 (56) | | 227 (60) |  |
|  |  | F | 40 (53) | 46 (61) | 45 (59) | 35 (46) | 30 (39) | 28 (37) | 30 (39) | 28 (37) | 23 (30) | 16 (21) | 168 (44) | | 153 (40) |  |
|  |  |  | χ2=1.0, ns | | χ2=2.6, ns | | χ2=0.1, ns | | χ2=0.1, ns | | χ2=1.7, ns | | | χ2=1.2, ns | | |
| Age  M(SD) | | | 26.8  (7.0) | 25.8  (8.1) | 26.0  (6.2) | 27.0  (6.8) | 29.8  (7.8) | 30.1  (8.9) | 28.1  (7.4) | 29.9  (9.5) | 27.3  (8.5) | 25.7  (6.2) | 27.6  (7.5) | | 27.7  (8.2) |  |
|  |  |  | t=-0.8, ns | | t=1.0, ns | | t=0.2, ns | | t=1.3, ns | | t=-1.3, ns | | | t=0.2, ns | | |

***2.2.*** ***Results***

## **sTable 4.** MNI coordinates and z scores for regions showing GMV changes in FEP relative to the HC using a template generated with a homogeneous sample.

| Region | | Peak MNI Coordinates (x,y,z) | Cluster Size  (No of voxels) | z | p |
| --- | --- | --- | --- | --- | --- |
| Decreased GVM in FEP relative to HC | | |  |  |  |
| L gyrus rectus | | -6,34,-21 | 123 | 9.6 | .003 |
| L orbital gyrus | | -3,42,-21 |  | 9.5 |  |
| R insula | | 37,-4,3 | 402 | 9.3 | <.001 |
| R thalamus | | 3,-20,3 | 430 | 9.1 | <.001 |
| R cuneus | | 3,-75,8 | 1295 | 8.8 | <.001 |
| R lingual gyrus | | 2,-78,-3 |  | 8.7 |  |
| L posterior cingulate | | 2,-68,14 |  | 8.7 |  |
| R inf. temporal gyrus | | 62,-32,-20 | 670 | 8.7 | <.001 |
| L sup. temporal pole | | -21,8,-32 | 154 | 8.7 | .002 |
| L fusiform gyrus | | -20,3,-40 |  | 8.6 |  |
| L gyrus rectus | | -4,2,-12 | 93 | 8.6 | .005 |
| L sup. temporal gyrus | | -50,16,-21 | 143 | 8.4 | .002 |
| L mid. temporal gyrus | | -58,-42,-16 | 176 | 7.2 | .001 |
| Increased GVM in FEP relative to HC | | |  |  |  |
| R cerebellum | 48,-70,-34 | | 292 | 8.7 | <.001 |
| R cerebellum | 24,-64,-46 | | 51 | 8.5 | .011 |
| L mid. cingulate gyrus | -12,-42,42 | | 174 | 8.3 | .001 |
| R cerebellum | 34,-39,-38 | | 196 | 8.1 | .001 |
| R sup. temporal gyrus | 34,6,-18 | | 52 | 8.1 | .01 |
| R precuneus | 12,-44,45 | | 140 | 8.0 | .002 |
| L cerebellum | -18,-56,-54 | | 91 | 7.6 | .005 |

GMV: grey matter volume; FEP: first episode psychosis; HC: healthy controls; L: left; R: right; med.: medial; mid.: middle; inf.: inferior; sup.: superior.

Overall, the results showed a very similar pattern frontal-temporal-insular regions of decreased GMV compared to the original VBM analysis. The clusters showing an increased GMV in FEP relative to HC were not as consistent and revealed further brain regions, including the cerebellum, cingulate and precuneus, in line with previous studies (Gao *et al.*, 2018; van Erp *et al.*, 2018).

# 3. Replication of the VBM analysis using a different size kernel

***3.1. Method***

To investigate the effect of the size of the smoothing kernel in the main results, the VBM analysis was repeated with a smaller 6mm and larger 10mm kernel. The images were pre-processed as described in section 2.3.2. of the main manuscript; however, using a smoothing of 6mm and 10mm kernel instead of 8mm. The results for each analysis are reported below.

***3.2. Results***

## **sTable 5.** MNI coordinates and z scores for regions showing GMV changes in FEP relative to the HC using a 6mm kernel.

| Region | | Peak MNI Coordinates (x,y,z) | Cluster Size  (No of voxels) | z | *p* |
| --- | --- | --- | --- | --- | --- |
| Decreased GVM in FEP relative to HC | | |  |  |  |
| L gyrus rectus | | 0,40,-22 | 117 | 9.7 | .001 |
| L med. orbital gyrus | | -8,54,-15 |  | 7.9 |  |
| L fusiform gyrus | | -20,2,-40 | 70 | 9.0 | .003 |
| L sup. temporal pole | | -22,10,-34 |  | 8.8 |  |
| R inf. temporal gyrus | | 51,-36,-26 | 328 | 8.8 | <.001 |
| L inf. temporal gyrus | | -48,-56,-26 | 179 | 8.8 | <.001 |
| L fusiform gyrus | | -42,-64,-21 |  | 8.2 |  |
| Increased GVM in FEP relative to HC | | |  |  |  |
| R sup. temporal gyrus | 38,18,-36 | | 196 | 8.2 | <.001 |
| R cerebellum | 44,-74,-34 | | 59 | 7.9 | .004 |

GMV: grey matter volume; FEP: first episode psychosis; HC: healthy controls; L: left; R: right; med.: medial; mid.: middle; inf.: inferior; sup.: superior.

Lowering the extended threshold to 25 consecutive voxels resulted in four additional clusters of decreased GMV in FEP (Table s4), all of which consistent with the original analysis: right lingual gyrus (MNI coordinates: 2,-82,-9, cluster size: 30, z=8.7 , *p*=.01) and left middle temporal gyrus (MNI coordinates: -62,-34,-12, cluster size: 30, z=8.0 , *p*=.01), superior temporal gyrus (MNI coordinates: -57,3,0; cluster size: 25, z=7.6 , *p*=0.012) and right insula gyrus (MNI coordinates: 41,17,-6, cluster size: 25, z=7.6, *p*=.015). An extended threshold to 25 consecutive voxels did not revealed any additional clusters of increased GMV in FEP relative to HC.

## **sTable 6.** MNI coordinates and z scores for regions showing GMV changes in FEP relative to the HC using a 10mm kernel.

| Region | | Peak MNI Coordinates (x,y,z) | Cluster Size  (No of voxels) | z | *p* |
| --- | --- | --- | --- | --- | --- |
| Decreased GVM in FEP relative to HC | | |  |  |  |
| L gyrus rectus | | -6,34,-22 | 237 | 9.7 | .003 |
| L sup. temporal pole | | -20,2,-40 | 144 | 9.4 | .007 |
| L fusiform gyrus | | -20,3,-42 |  | 8.9 |  |
| R inf. temporal gyrus | | 54,-32,-21 | 876 | 9.3 | <.001 |
| R mid. temporal gyrus | | 58,-27,-12 |  | 9.1 |  |
| L mid. temporal gyrus | | -57,-22,-15 | 756 | 9.1 | <.001 |
| L inf. orbital gyrus | | -48,15,-9 | 354 | 8.7 | .001 |
| L sup. temporal pole | | -48,20,-18 |  | 8.5 |  |
| R insula | | 45,18,-10 | 318 | 8.7 | .002 |
| R inf. orbital gyrus | | 46,24,-16 |  | 8.4 |  |
| R lingual gyrus | | 3,-81,-12 | 80 | 8.6 | .013 |
| Increased GVM in FEP relative to HC | | |  |  |  |
| R sup. temporal gyrus | 39,15,-36 | | 444 | 8.0 | .001 |

GMV: grey matter volume; FEP: first episode psychosis; HC: healthy controls; L: left; R: right; med.: medial; mid.: middle; inf.: inferior; sup.: superior.

The use of a 10mm smoothing kernel also resulted in a very similar pattern of results compared to the main VBM analysis, both in regions displaying a decreased and increased in GMV.

#
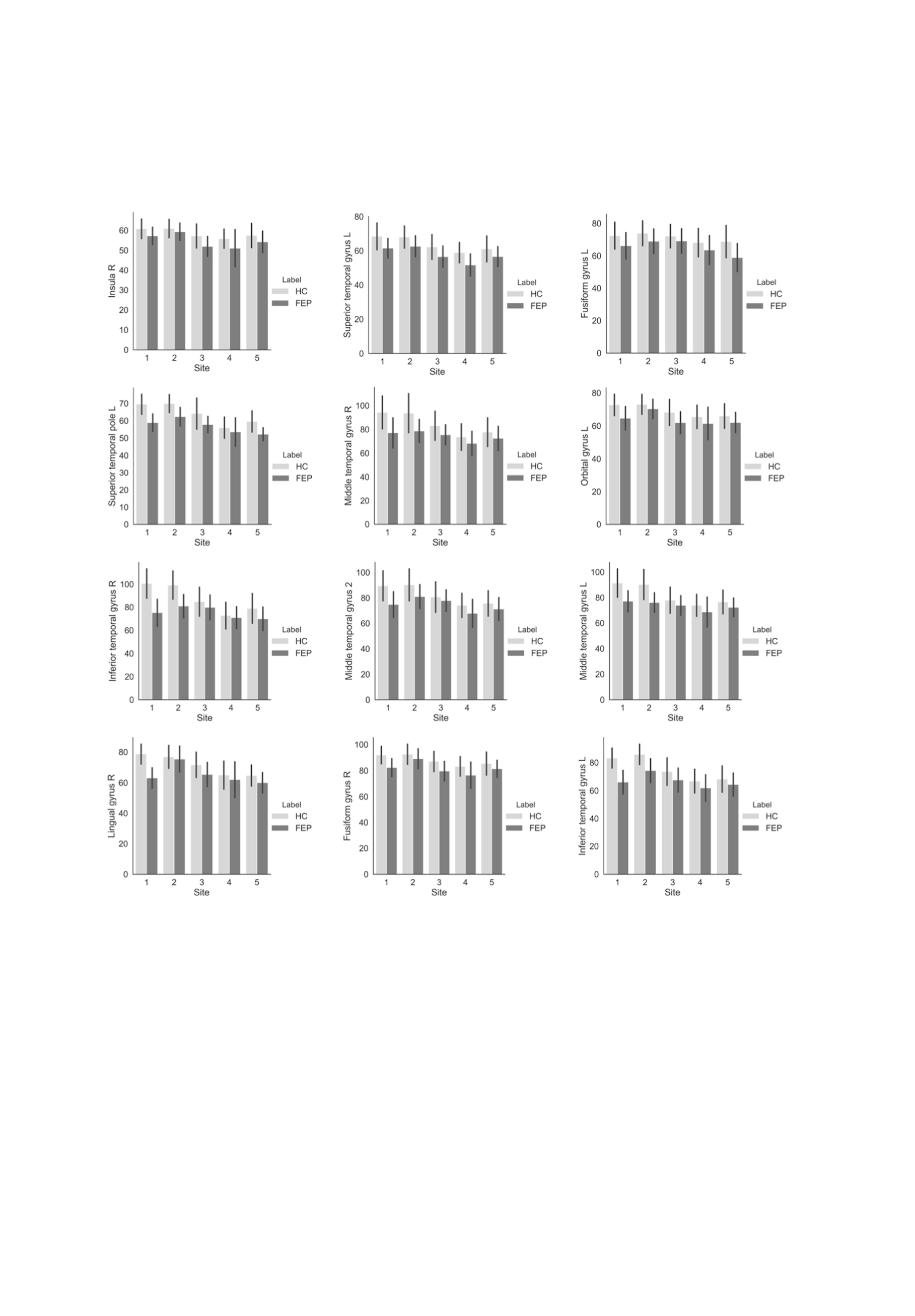
sFigure 1. Mean-plots for the GMV of the peak coordinates of each cluster (error bars represent standard deviation).

**sFigure 2.** Scatter plots for the significant correlations in Table 4.

**
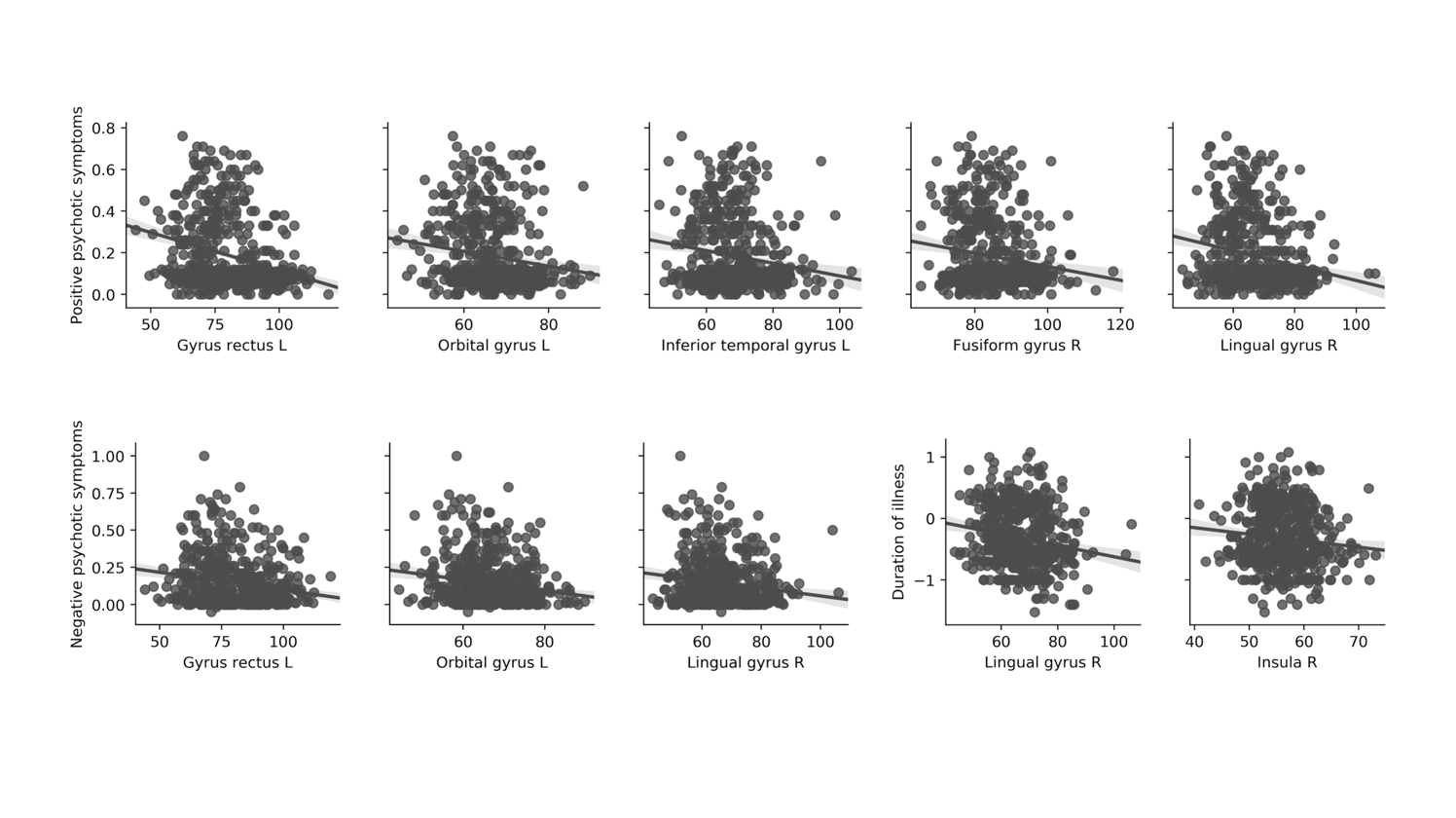
**
